# Systemic inhibition of 5-lipoxygenase by MK-886 exacerbates apical periodontitis bone loss in a mouse model

**DOI:** 10.1186/s12903-023-02712-w

**Published:** 2023-01-10

**Authors:** Igor Bassi Ferreira Petean, Alice Corrêa Silva-Sousa, Raquel Assed Bezerra da Silva, Marília Pacífico Lucisano, Léa Assed Bezerra da Silva, Guilherme Piedade Assed de Castro, Manoel Damião Sousa-Neto, Lúcia Helena Faccioli, Francisco Wanderley Garcia Paula-Silva

**Affiliations:** 1 School of Dentistry of Ribeirão Preto, University of São Paulo, Avenida do Café, s/n, Ribeirão Preto, São Paulo Brazil; 2 Faculdade de Ciências Farmacêuticas de Ribeirão Preto, Universidade de São Paulo, Ribeirão Preto, São Paulo Brazil

**Keywords:** Apical periodontitis, Bacterial lipopolysaccharide, 5-Lipoxygenase, MK-886, In vivo model

## Abstract

**Background:**

To investigate if 5-LO selective inhibitor (MK-886) could be used for systemic treatment of experimentally induced apical periodontitis in a mouse model.

**Methods:**

Twenty-four C57BL/6 mice were used. After coronal opening, a solution containing *Escherichia* *coli* LPS (1.0 µg/µL) was inoculated into the root canals of the lower and upper right first molars (n = 72 teeth). After 30 days apical periodontitis was established, and the animals were treated with MK-886 (5 mg/kg), a 5-LO inhibitor, for 7 and 14 days. The tissues were removed for histopathological and histometric analyses, evaluation of osteoclast number and gene expression for receptor activator of nuclear factor kappa-B (*Tnfrsf11a*), receptor activator of nuclear factor kappa-B ligand (*Tnfsf11*), osteoprotegerin (*Tnfrsf11b)*, tartrate-resistant acid phosphatase (*Acp5*), matrix metalloproteinase-9 (*Mmp9*), cathepsin K (*Ctsk*) and calcitonin receptor (*Calcr*)*.* Statistical data analysis was performed using Kruskal Wallis followed by Dunn’s tests (α = 0.05).

**Results:**

Administration of MK-886 for 7 days exerted no effect on apical periodontitis progression compared to LPS inoculation without treatment (*p* = 0.3549), while treatment for 14 days exacerbated bone loss (*p* < 0.0001). Administration of MK-886 enhanced osteoclastogenesis signaling and osteoclast formation within 7 days (*p* = 0.0005), but exerted no effect at 14 days (*p* > 0.9999). After 7 days of treatment, MK-886 induced mRNA expression for *Acp5* (*p* = 0.0001), *Calcr* (*p* = 0.0003), *Mmp9* (*p* = 0.0005) and *Ctsk* (*p* = 0.0008), however no effect in those gene expression was observed after 14 days (*p* > 0.05).

**Conclusion:**

Systemic treatment with MK-886 exacerbated LPS-induced apical periodontitis in a mouse model.

## Background

Apical periodontitis (AP) is a localized immune response against endodontic infection, a sequel of the host defense response to the microbial challenge that cause dental and bone resorption of apical and periapical tissues [1–3]. Recently, a meta-analysis confirmed a high global prevalence of AP, with 52% of pooled samples worldwide reporting at least one tooth with AP [3]. Thus, reduction of the bacteria load and their toxins and allow the host response to proceed with repair to complete healing of the periapical pathosis and the restoration of the function are the ultimate goals of non-surgical root canal therapy (NSRCT) [1, 2, 4]. However, fail of NSRCT may occur in up to 32% in cases of teeth with AP [5, 6].

Considering that AP is a multifactorial disease, to reduce failure in the NSRCT approaches that address microbiology, quality of treatment and host response particularities are needed [7]. Because AP is a host tissue inflammatory response to bacterial infection of the root canal and serves the purpose of restraining the progression of infection towards bone tissue, systemic therapy would be useful in cases of difficult healing apical periodontitis such as lesions in patients with comorbidities or dysbiosis. Thus, recent research investigates coadjutant therapies to limit the AP development and establishment, or improve their healing rates after NSRCT, with promising results through the use of anti-inflammatory drugs and probiotics via systemic or topical administration, in order to control the inflammatory process during AP establishment and repair [8–11].

The inflammatory response in apical periodontitis involves the generation of eicosanoids, synthesized from the metabolization of arachidonic acid by cyclooxygenase (COX) or lipoxygenase (LO) enzymes [2, 12–15]. Leukotrienes (LT), a class of metabolites generated downstream of 5-LO and 5-LO activating protein (FLAP) [16] have been found in rat inflamed dental pulp and human AP [17–19]. The enzyme 5-lipoxygenase (5-LO) and its metabolites stimulate the formation and activity of osteoclasts in vitro [20, 21], however, 5-LO gene ablation resulted in increased bone loss in cases of apical periodontitis when root canals were infected with Gram-negative *Fusobacterium nucleatum* [22] or it had no impact on the enlargement of apical periodontitis, when root canals were infected with micro-organisms from oral cavity [15]. Bone metabolism is regulated by receptor activator of nuclear factor kappa-B (RANK), receptor activator of nuclear factor kappa-B ligand (RANKL) and osteoprotegerin (OPG). Resorption occurs when there is an imbalance in the expression of RANKL and OPG, due to inflammatory conditions that result in increase in RANKL activity and decrease in the regulatory activity of OPG [2, 23].

Although it is mainly used for asthma treatment, MK-886 is a 5-lipoxygenase activating protein inhibitor can be a pharmacological strategy for intervention in leukotriene biosynthesis to control inflammatory diseases [24, 25]. Because 5-LO inhibition with CJ-13610 attenuated inflammation and bone loss in lipopolysaccharide (LPS)-induced periodontal disease [26] and we previously found that short term inhibition of 5-LO resulted in reduced inflammatory mediator and RANKL synthesis during AP development [12], we sought to investigate the effects of systemic administration of MK-886 for 7 and 14 days in mature AP. Since impairment of catabolic signaling could be a therapeutic measure to prevent bone loss in cases of apical periodontitis, the aim of this study was to investigate whether 5-LO inhibitor (MK-886) could be used as systemic treatment of experimentally induced apical periodontitis in mice. The null hypothesis of this study was that 5-LO inhibition would not prevent bone resorption in apical periodontitis.

## Methods

### Animals

This research was conducted in accordance with Brazilian Guideline for the Care and Use of Animals in Teaching or Scientific Research Activities regulated by the National Council for the Control of Animal experimentation (Law 11.794/2008), conformed to Animal Research: Reporting of In Vivo Experiments (ARRIVE) guidelines and the animal study protocol was approved by the Ethical Committee at the University of São Paulo (IRB approval number 12.1.60.53.8). C57BL/6 male mice (n = 24) (*Mus musculus*), 8-week-old, weighting 18-20 g, were used for this study. The sample size was determined according to a previous study [10]. For the operative procedures, animals were anaesthetized i.m. with 10% ketamine hydrochloride (150 mg/kg; National Pharmaceutical Chemistry Union Agener S/A, Embu-Guaçu, SP, Brazil) and xylazine (7.5 mg/kg; Dopaser, Labs Calier S/A, Barcelona, Spain). Anaesthesia was sustained during the operative procedures throughout the experimental period, and the animals were monitored by a veterinarian throughout the entire experimental period.

### LPS inoculation for induction of apical periodontitis

A surgical table with a device for mandibular retraction was used to keep the animals immobilized with mouth open for direct access to the molars. The mandibular and maxillary right first molars of each animal were used for LPS inoculation into the root canals. The first molars of the left side were not inoculated and served as the negative control group. Access cavity preparations were performed with 1011 spherical diamond burs (KG Sorensen Ind. Com. Ltda., Barueri, SP, Brazil) and 10 μL of LPS suspension (1.0 µg/µL) of *E. coli* 0127: B8 (L3129; Sigma-Aldrich Corp., St. Louis, MO, USA) were inoculated into the root canals of each tooth with use of an automatic micropipette [10–15]. Conventional glass ionomer cement (S.S. White Dental Articles Ltda, Rio de Janeiro, RJ, Brazil), manipulated according to manufacturer’s instructions, was used for restoring the teeth. After LPS inoculation, the animals were kept under supervision for thirty days, in order to establish the apical periodontitis.

### Systemic drug treatment

**Fig. 1:**
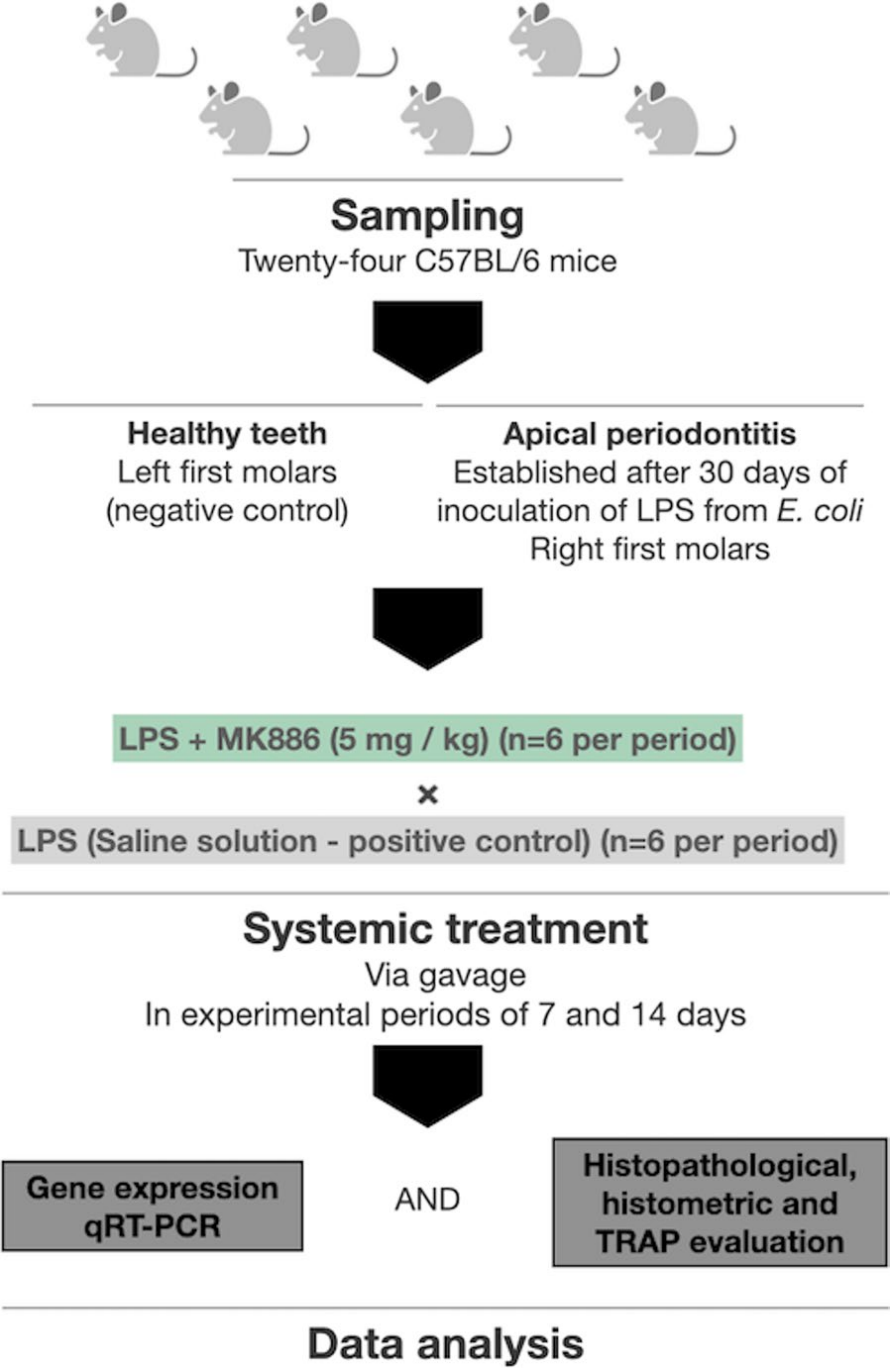
Flow diagram of the study design

After apical periodontitis was established, the animals were treated with MK-886 (Merck Millipore, Darmstadt, Germany). MK-886 was dissolved in ethanol (100 µL), according to manufacturer’s recommendations, diluted in distilled water (400 µL), and provided by gavage (0.5 mL, 5 mg/kg body weight) daily for time intervals of 7 and 14 days. Healthy teeth from animals on the opposite side that did not receive medication were used as negative control and teeth from group which the pulp chamber filled with LPS without MK-886 treatment was used as a positive control. The groups were determined by inoculation with LPS (n = 6 teeth for each experimental time interval), LPS + MK-886 (n = 6 teeth for each experimental time interval). At time intervals of 7 and 14 days after starting with treatment, the animals were euthanized by means of anesthetic overdose and the tissues were collected. The Fig. 1 is a visual representation of the group distribution and study design.

### Histological preparation

The upper and lower jaws were dissected and removed using surgical scissors. The blocks containing tooth and bone were fixed in 10% buffered formalin for 24 h at room temperature and demineralized in 10% EDTA (Merck S.A. Chemical Industries, Rio de Janeiro, RJ, Brazil) for approximately 21 days. After demineralization, the blocks of specimens were submitted to routine histological processing, washed in running water for 24 h, dehydrated in increasing concentrations of alcohol, diaphanized in xylol, and embedded in paraffin. The blocks were sectioned longitudinally, in the bucco-lingual direction, to obtain slices with a thickness of 5 µm. Sections were stained with haematoxylin and eosin (HE) for histopathological and histometric evaluation and submitted to tartrate-resistant acid phosphatase (TRAP) enzyme assay, for enzyme histochemical analysis.

### Morphometric analysis of AP size under light microscopy

Longitudinal sections of the teeth were obtained and stained by haematoxylin–eosin. The analysis was qualitatively performed by using conventional light microscopy (Zeiss Axio Imager, Carl Zeiss AG Light Microscopy, Göttingen, Germany). All the analyses were performed using videomicroscopy with the Zeiss AxioVision Software (Carl Zeiss AG Light Microscopy), in conjunction with the AxioCam MRc5 (Carl Zeiss AG Light Microscopy) microscope and camcorder, at 20× magnification, operated in fluorescence mode (excitation at 460–500 nm and emission at 512–542 nm). For each specimen, the area of apical periodontitis was delineated and measured in mm^2^ on sections representing the largest diameter of the lesion, using the ImageJ 1.50i software (NIH, Bethesda, MD). The delineation of the lesion excluded intact structures (periodontal ligament, cementum and alveolar bone), and included areas of resorption and inflammatory infiltrate, according to previously described parameters [10].”

### Determination of the activity and presence of osteoclasts by enzyme histochemical analyses for TRAP

The analysis of activity and the number of positive osteoclasts were analyzed and determined by counting the number of multinucleate TRAP-positive cells in the resorption lacunae in direct contact with the alveolar bone around the area of apical periodontitis. The tissue sections were deparaffinized and incubated in a solution containing 8 mg of naphthol AS-MX di-sodium phosphate (Sigma-Aldrich) in 500 μL of NN-dimethylformamide. This was followed by the addition of 50 mL of a 0.2 mol buffer solution/L sodium acetate (pH 5.0) containing 70 mg of Fast Red ITR (Sigma-Aldrich). Next, the sodium tartrate substrate dihydrate (50 mmol/L) was added to the solution and incubated at 37 °C for 12 h. After this, the sections were washed in distilled water and stained with hematoxylin. Other sections were incubated with a medium without substrate as a control.

### Quantitative reverse transcriptase-polymerase chain reaction (qRT-PCR)

The maxillary right first molars of three animals were used for the Experimental Group and their maxillary left first molars, for the Control Group. RNA extraction was performed with RNeasy Mini kit (RNeasy^®^ Mini, Qiagen Inc., Valencia, CA, USA) and samples were treated with DNAse I (RNase-Free DNase Set; Qiagen Inc.), according to manufacturer’s protocol. RNA integrity was analyzed using 1% agarose electrophoresis and quantity was estimated in NanoDrop 1000 (Thermo Fisher Scientific Inc., Wilmington, DE, USA) at 230, 260 and 280 ηm wavelengths.

From 2000 ng of total RNA using random primers (High Quality cDNA Reverse Transcriptase Kits, Applied Biosystems, Foster City, CA, USA) complimentary DNA (cDNA) was synthesized. Aliquots of 2 µL of the total cDNA were amplified by qRT-PCR using primers for *Tnfrsf11a* (RANK; Mm00437135), *Tnfsf11* (RANKL; Mm00441906), *Tnfrsf11b* (OPG; Mm01205928), *Acp5* (Mm00475698), *Mmp9* (Mm00442991), *Ctsk* (Mm00484039) and *Calcr* (Mm01197736) (TaqMan^®^ Gene Expression Assay, Applied Biosystems) in an StepOne Plus equipment (Applied Biosystems). *Gapdh* (Mm99999915) was used as reference gene. Duplicates were used for qRT-PCR reactions and amplification was done with denaturation at 95 °C for 2 min; followed by 40 cycles of 95 °C for 1 s and 60 °C for 20 s. Relative quantification was performed using the ΔΔCt Method.

### Statistical analysis

Data were analyzed using GraphPad Prism 8.0 (GraphPad, San Diego, CA) and presented non normal distribution, therefore statistical analyses were performed using nonparametric Kruskal Wallis followed by Dunn's tests (α = 0.05).

## Results

### Administration of MK-886 favored apical periodontitis bone loss

**Fig. 2:**
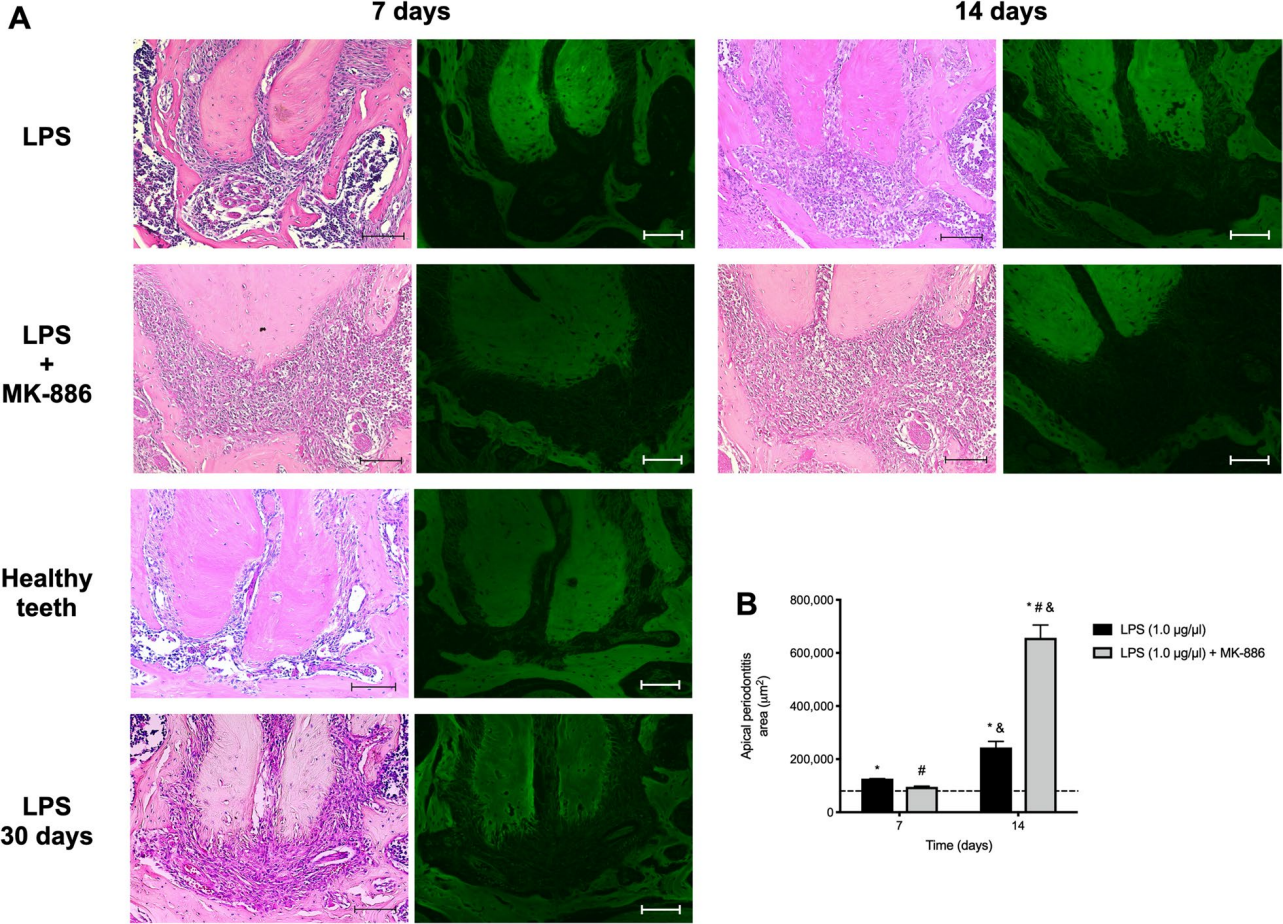
Apical periodontitis establishment and status after treatment with MK-886. **A** Photomicrographs of healthy teeth, obtained after LPS inoculation into root canals, or at time intervals of 7 and 14 days after treatment with and without MK-886. HE, original 20× magnification (scale bar = 100 µm). **B** Graphic representation of the measurement of area of apical periodontitis, in µm^2^ in the different groups at 7 and 14 days (median and interquartile range). Dashed line indicates the area of a healthy periodontal ligament. * *p* < 0.05 compared with healthy periodontal ligament; # *p* < 0.05 compared with inoculation of LPS alone into the root canals; &*p* < 0.05 comparison between 7 and 14 days of the same treatment

Thirty days after inoculation of LPS into the root canals, apical periodontitis was formed and was characterized by a wide periodontal ligament space, increased recruitment of inflammatory cells and bone loss. Treatment with MK-886 for 7 days had no effect on the progression of apical periodontitis when compared with LPS inoculation without any treatment (*p* = 0.3549), while treatment for 14 days significantly exacerbated bone loss in apical periodontitis (*p* < 0.0001) (Fig. 2).

### MK-886 treatment increased periapical osteoclastogenesis signaling and osteoclast formation

**Fig. 3:**
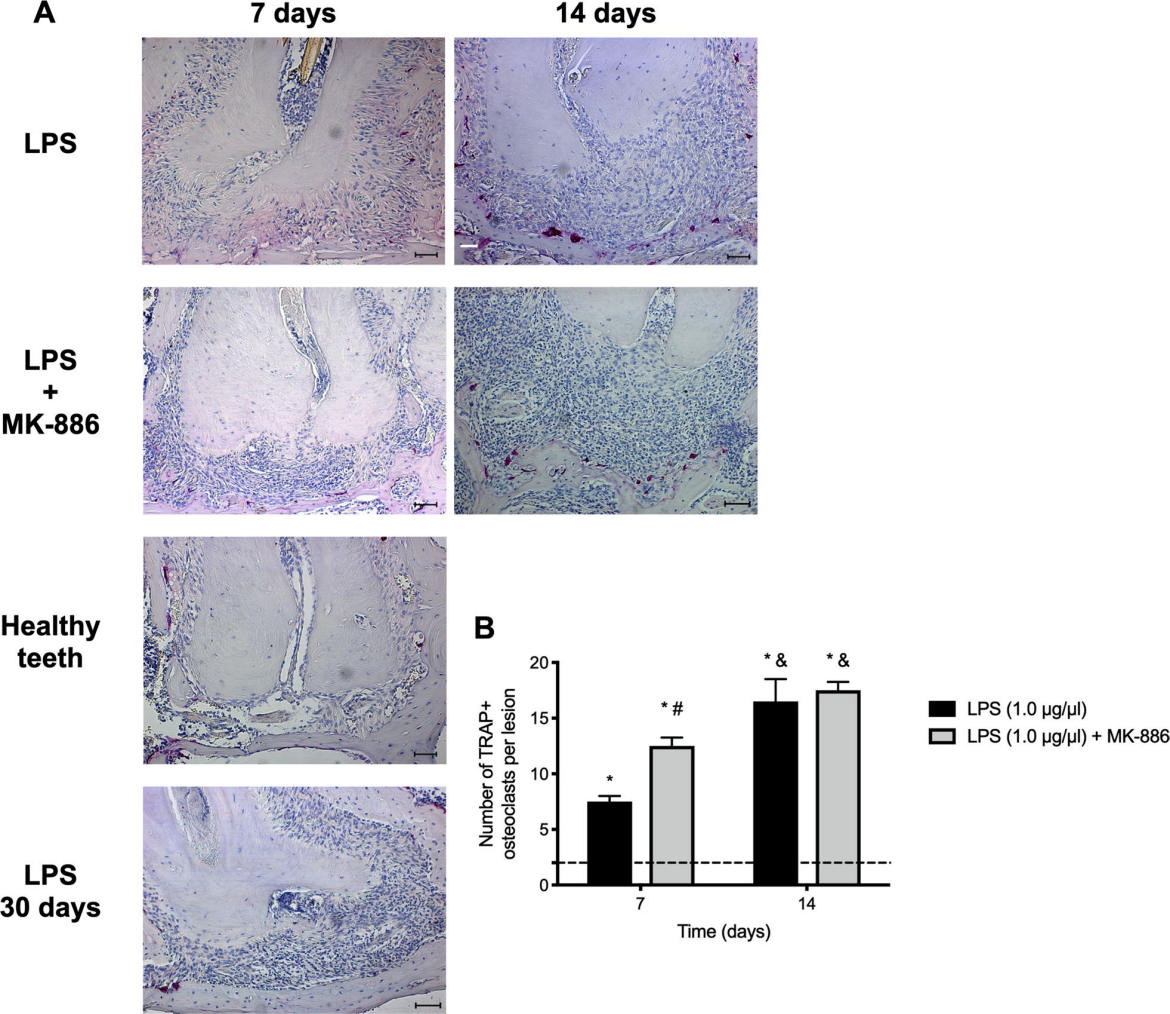
Osteoclast formation after treatment with MK-886. **A** Photomicrographs representative of enzyme histochemical findings for the tartrate-resistant acid phosphatase enzyme (TRAP) obtained from healthy teeth, after LPS inoculation into the root canals, or after 7 and 14 days of treatment with MK-886. Original magnification 20 × (scale bar = 100 µm). **B** Graphic representation of the number of tartrate-resistant acid phosphatase enzyme (TRAP) positive osteoclasts per lesion in the different groups at 7 and 14 days (median and interquartile range). Dashed line indicates number of TRAP + osteoclasts in healthy apical periodontal ligament. *p < 0.05 compared with healthy periodontal ligament; #*p* < 0.05 compared with inoculation of LPS alone into the root canals; &*p* < 0.05 comparison between 7 and 14 days of the same treatment

Significant increase in the number of osteoclasts was observed after LPS inoculation into root canals, when compared with healthy teeth (*p* < 0.0001). Treatment with MK-886 for 7 days augmented osteoclast formation significantly (*p* = 0.0005) but had no effect at 14 days when compared with LPS inoculation without any treatment (*p* > 0.9999) (Fig. 3).

**Fig. 4:**
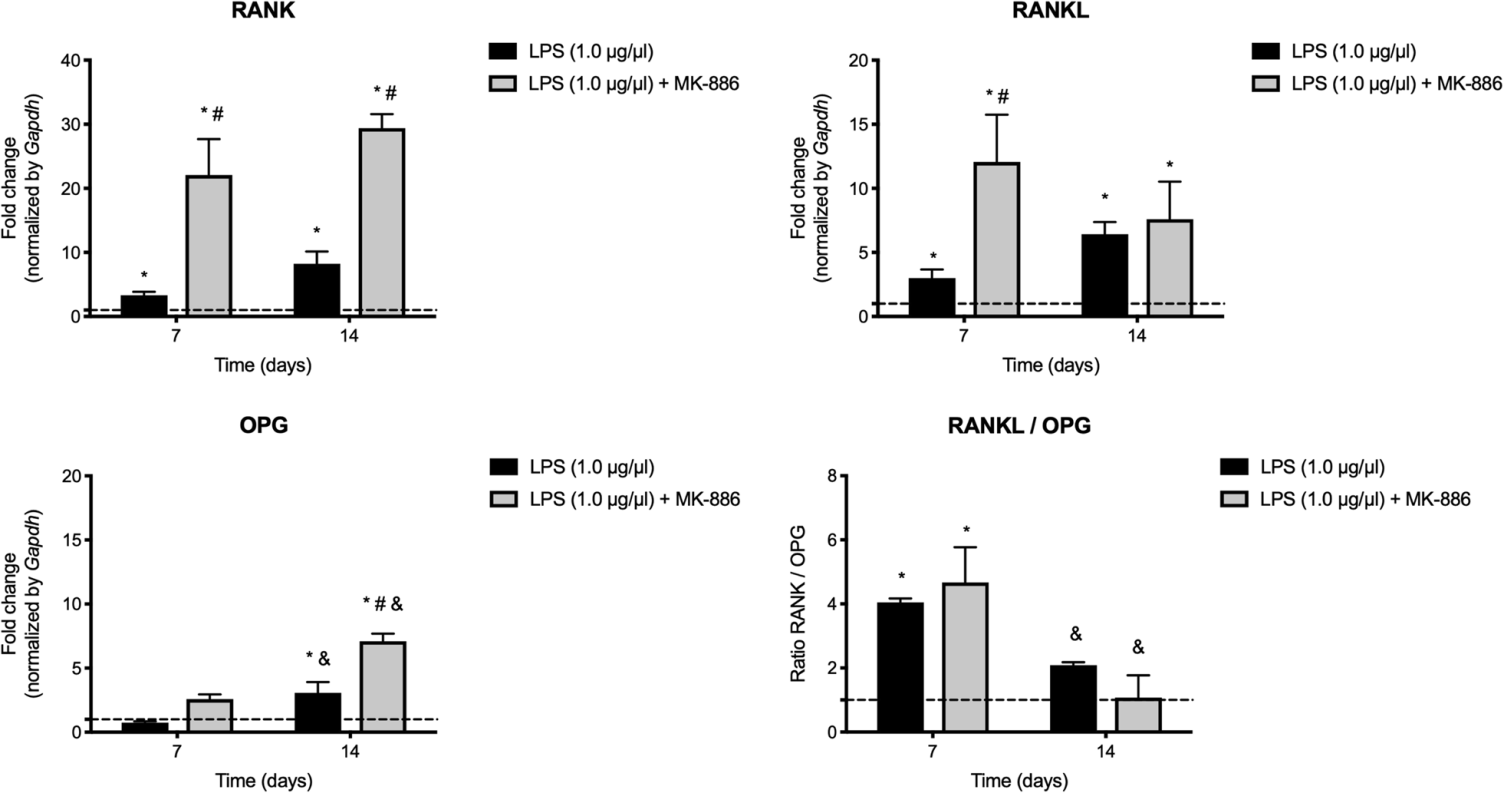
Detection of RANK/RANKL/OPG genes after treatment with MK-886. Relative expression of *Tnfrsf11a*, *Tnfsf11* and *Tnfrsf11b* genes that encode RANK, RANKL and OPG, after inoculation of LPS into root canals and at 7 and 14 days of treatment with MK-886 (median and interquartile range). *p < 0.05 compared with healthy periodontal ligament; #*p* < 0.05 compared with inoculation of LPS into root canals; &*p* < 0.05 comparison between 7 and 14 days of the same treatment

The effect of MK-886, with regard to the expression of RANK, RANKL and OPG revealed that this administration induced significantly higher RANK expression when compared with LPS inoculation without any treatment, after both at 7 and 14 days (*p* = 0.0027 and *p* = 0.0014, respectively). Furthermore, treatment for 7 days induced significant RANKL expression (*p* = 0.0278) without effect at 14 days (*p* = 0.9877); while MK-886 significantly upregulated OPG after both at 7 and 14 days (*p* = 0,0481 and *p* = 0.0009, respectively). Higher expression of RANKL over OPG (Ratio > 1) was found at 7 days for LPS and LPS + MK-886 treatment (*p* = 0.0099 and *p* = 0.0038, respectively), without significant difference between them (*p* = 0.8462). No catabolic over anabolic signaling was detected at 14 days (Fig. 4).

### Treatment with MK-886 stimulated early expression of genes involved in bone resorption

**Fig. 5:**
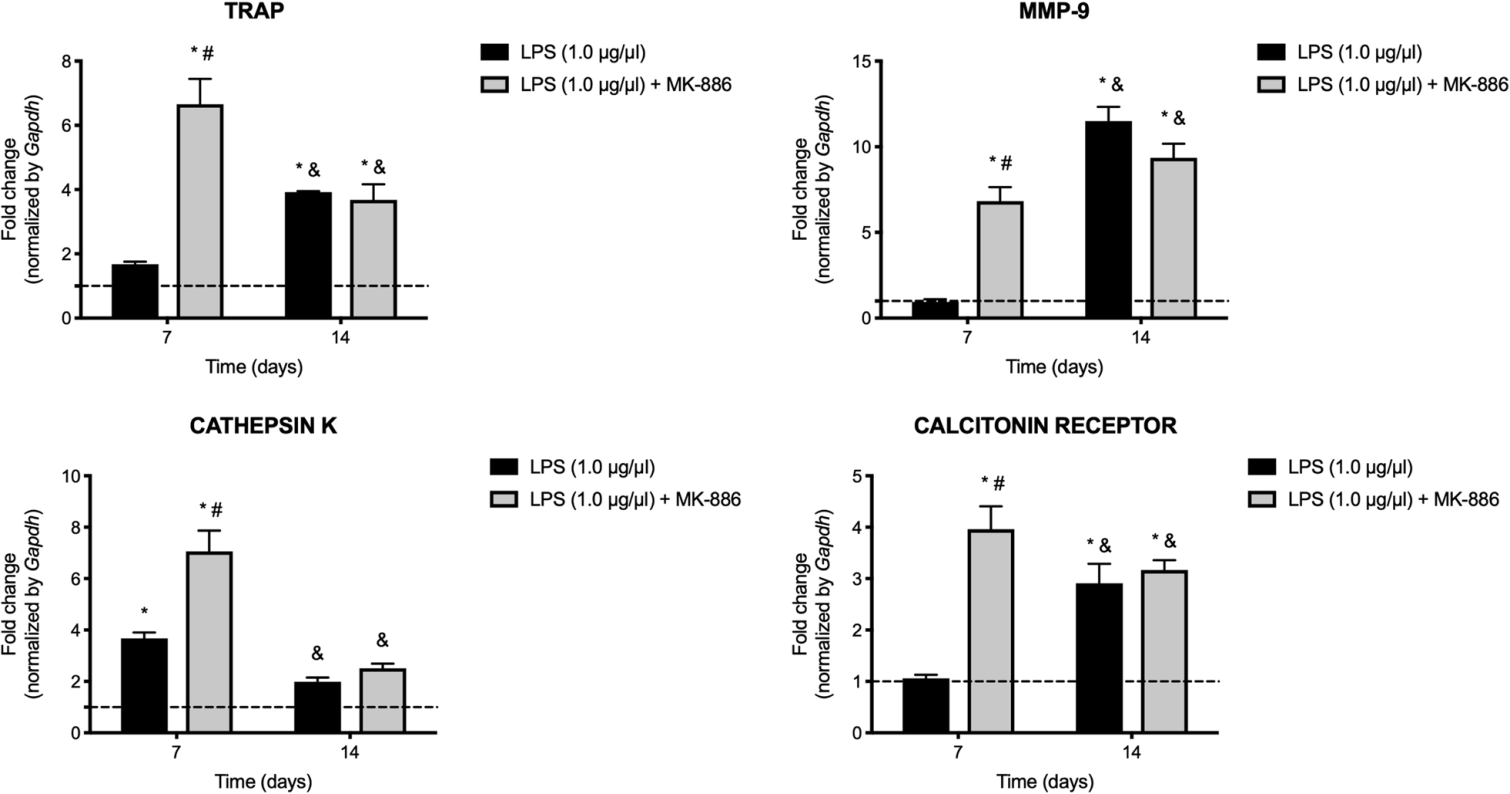
Detection of genes related to bone metabolism after treatment with MK-886. Relative expression of *Acp5*, *Mmp9, Ctsk* and *Calcr* genes that encode the tartrate-resistant acid phosphatase enzyme (TRAP), matrix-9 metalloproteinase, cathepsin K and the calcitonin receptor, after LPS inoculation into root canals, and at 7 and 14 days after treatment with MK-886 (median and interquartile range). *p < 0.05 compared with healthy periodontal ligament; #*p* < 0.05 compared with inoculation of LPS into the root canals; &*p* < 0.05 comparison between 7 and 14 days of the same treatment

After 7 days of treatment, MK-886 induced significant mRNA expression of the enzymes TRAP (*p* = 0.0001), MMP-9 (*p* = 0.0005), cathepsin K (*p* = 0.0008) and for calcitonin receptor (*p* = 0.0003). After 14 days, TRAP, MMP-9 and calcitonin receptor expression induced after inoculation of LPS into the root canals was not modulated by treatment with MK-886 (*p* = 0.9849, *p* = 0.0746 and *p* = 0.9185, respectively). Cathepsin K mRNA expression did not change as a result of LPS inoculation alone and after treatment with MK-886 (*p* = 0.2095 and *p* = 0.7240, respectively) (Fig. 5).

## Discussion

In this study it was found that treatment of apical periodontitis using 5-LO inhibitor MK-866 for 7 days enhanced osteoclast signaling and formation and treatment for 14 days increased bone loss and resulted in larger apical periodontitis lesions. The null hypothesis of this study was therefore rejected.

Previous investigations have reported that at 28 days, periapical bone resorption occurs after inoculation of 10 µL of LPS suspension into the root canals [10, 11]. Therefore, in the present study, the period of 30 days after inoculation of LPS was investigated, which meant that apical periodontitis was established prior to the treatment with MK-886. Furthermore, data of the size and morphological aspects of the area of apical periodontitis were evaluated at 30 days, when tissue necrosis, thickening of the periodontal space and recruitment of inflammatory mediators were observed, confirming the establishment of apical periodontitis.

Regarding to the results, it has previously been demonstrated that 5-lipoxygenase inhibition plays a dual-role in osteoclastogenesis during bacteria-induced AP formation. Early on (7 and 14 days), osteoclastogenesis signaling was down-regulated by the inhibition of 5-LO, but in the long-term (21 and 28 days) inhibition failed to prevent synthesis of catabolic mediators that resulted in increased bone loss [12]. Here, treatment with 5-LO inhibitor in an already established LPS-induced AP lesion, exacerbated the catabolic effects on bone. Although the experimental strategy was different from this study in which we used LPS inoculation to promote a mature and stablished apical periodontitis, the observed behavior in osteoclast signaling was similar to that observed previously [12]. MK-886 treatment stimulated RANKL production at 21 days which was stabilized at 28 days, after the establishment of a mature apical periodontitis developed after root canal contamination from oral environment. It is worth mentioning that the osteoclastogenesis signaling was not coincident with the presence of active osteoclasts, once this is a dynamic process. Thus, we observed that initially (7 days) there was an overexpression of these genes, that resulted in a more robust osteoclastogenesis and consequently, bone resorption, which tends to reach a limit, that reflects in the levels of expression of genes that regulates this process after 14 days of treatment.

One possible explanation of these catabolic effects on bone after treatment with MK-886, relies on the crucial role of leukotrienes as efficient chemotactic agents, which promote polymorphonuclear aggregation and degranulation and stimulate leukocyte adherence to the endothelial wall for transmigration of inflammatory cells [27]. Inhibition of synthesis of 5-LO metabolites might have impaired the immune and inflammatory cell recruitment and response that resulted in augmented bone resorption. Indeed, an impaired host innate immune system and severe AP were found in an experimental 5-LO knockout murine model [22]. Moreover, the 5-LO pathway plays a critical role in the stimulation of inflammatory mediator synthesis in polymicrobial-induced apical periodontitis in mice [15].

In vivo inhibition or ablation of 5-LO has been shown to have controversial effects on several bone diseases, i.e., this may have anabolic [12, 14, 22] or catabolic [25, 28] effects. In vitro*,* the mechanism involved in osteoclast formation induced by 5-LO has been demonstrated [28, 29]. Leukotrienes B_4_ (LTB_4_) and C_4_ (LTC_4_), metabolites of 5-LO, induce RANKL and osteoclast differentiation of bone marrow-derived and RAW-264.7 macrophages [20, 21]. In a previous in vitro study, we found that 5-LO pathway is involved in the osteoclastic differentiation hematopoietic lineage cells and that exogenous addition of LTB_4_, loaded in microspheres (MS) inhibited osteoclastogenesis induced by M-CSF and RANKL [30]. The mechanism  involved induction of *MMP9* gene expression and inhibition of *CALCR* and *CTSK*, without changing *ACP5* [30]. Furthermore, 5-LO activity may be modulated by nitric oxide (NO) since prolonged exposure to lipopolysaccharide inhibits macrophage 5-LO metabolism by induction of NO synthesis [31]. In addition, increased generation of hydrogen peroxide (H_2_O_2_) and an inhibited production of NO were observed after in-vitro incubation of MK-886 with peritoneal macrophages, which have been related to the replication of infectious agents [32]. Therefore, one possible explanation for the divergent effects observed here could be the fact that LTB_4_ enhances phagocytosis and killing of microorganisms, and its long-term inhibition might have impaired infection control resulting in increased bone loss.

With regards to undesirable systemic effects of 5-LO inhibitor administration, according to the literature these drugs are generally well-tolerated, and the majority of adverse events described were considered mild (headache, gastrointestinal disorders, pharyngitis, fatigue, upper respiratory tract infection, cutaneous rash and reversible alterations in levels of serum transaminase) [33].

Although the main goal of root canal treatment is widely known to be to reduce the bacteria load and their by-products that contribute to the perpetuation of apical periodontitis [34, 35], promising results have been shown with the use of anti-inflammatory drugs and probiotics administered systemically or topically, and have demonstrated an advance in accelerating bone healing or preventing bone loss in apical periodontitis [8–10]. In this study, a histological and histometric evaluation was performed to understand the signaling pathways and mediators involved in bone resorption. This method is commonly used to determine periapical bone loss in induced apical periodontitis [10, 12] but this analysis could be improved through tomographic volumetric measurements [36–38]. This is one limitation of the present study, which should be better explored in future studies. Another limitation is regarding to the animal model, once the use of a mouse model where the root canal treatment was not performed may have impacted in the results observed, considering that the persistence of infection contributes to apical periodontitis development [1]. Moreover, in the search for the ideal medication to control and reverse the apical periodontitis, the positive and negative aspects of the medicaments used should be evaluated, considering that it impacts on the choice of medicament to be used in routine clinical procedures.

Recently, Petean et al. (2021) [10] evaluated the efficacy of systemic use of selective and non-selective inhibitors of cyclooxygenase-2 enzymes in the treatment of experimental apical periodontitis induced by bacterial lipopolysaccharide (LPS) in vivo in a mouse model, and the results demonstrated promising results with the use of COX-2 inhibitor Celecoxib, which dampened osteoclastogenic signaling, and activity that suppressed bone resorption. Nevertheless, here it was demonstrated that inhibition of 5-LO by MK-886 is not suitable as a coadjutant systemic treatment in clinical practice to prevent bone resorption in AP.

## Conclusions

Systemic treatment with MK-886 exacerbated LPS-induced apical periodontitis in a murine model. Inhibition of 5-LO was, therefore, not a successful strategy to prevent bone resorption in apical periodontitis.

## Data Availability

All the data generated during the study are available in the manuscript itself.
